# The density of doublecortin cells in the piriform cortex is affected by transition to adulthood but not first pregnancy in mice

**DOI:** 10.1007/s00429-025-02957-x

**Published:** 2025-06-10

**Authors:** Rafael Esteve-Pérez, María Abellán-Álvaro, Cinta Navarro-Moreno, Michele Prina, Manuela Barneo-Muñoz, Enrique Lanuza, María José Sánchez-Catalán, Fernando Martínez-García, Jose Vicente Torres-Pérez, Carmen Agustín-Pavón

**Affiliations:** 1 Departament de Biologia Cel·lular, Biologia Funcional i Antropologia Física, Facultat de Ciències Biològiques, Universitat de València, C/ Dr Moliner, 50, 46100 Valencia, Spain; 2 Departament de Medicina, Universitat Jaume I, Castelló de la Plana, Spain; 3 Department of Biomedical Sciences, Faculty of Health Sciences, Institute of Biomedical Sciences, Cardenal Herrera-CEU University, Valencia, Spain; 4 Present Address: CIBERER - IIS La Fe, Valencia, Spain

**Keywords:** Adolescence, Matrescence, Neurogenesis, Piriform cortex, Motherhood, Doublecortin

## Abstract

Motherhood is a critical period modulating behavioural changes to favour survival in mammals. In mice, olfaction is a key driver of social behaviours, and adult neurogenesis in the olfactory bulb is modulated at this stage, contributing to pup recognition. The primary olfactory cortex, known as the piriform cortex, contains a special population of neurons of embryonic origin which continues expressing immature markers such as doublecortin (DCX) in young and adult mice, and present a long period of protracted maturation and integration into the circuit as the animal ages. While these immature neurons are considered a potential source of plasticity, their precise role and whether motherhood affects their maturation rate remains unclear. To investigate this question, we analysed the expression of DCX in prepubescent vs. young adults, in virgin vs. pregnant and in pup-sensitized virgins vs. lactating female mice. We found that the density of DCX cells sharply decreased in the piriform cortex with age, previously demonstrated as a proxy of their integration as glutamatergic neurons by contrast, pregnancy and lactation failed to significantly alter the density of these cells. To further analyse how motherhood could affect DCX-ir cells, we co-labelled DCX cells with NeuN, an archetypical marker of mature neurons. We did not find significant differences in the percentage of double-labelled cells nor in features related to maturation like number of neurites or main diameter in lactating dams as compared to pup-sensitized virgin females. Our results suggest that the first pregnancy does not significantly affect the differentiation of immature neurons of the piriform cortex.

## Introduction

Throughout the lifespan of a female mammal, the transition to adulthood and motherhood constitute periods of special importance due to its adaptive nature (Boivin et al. 2017; Carmona et al. 2019; Pawluski et al. 2022; Hoekzema et al. 2022). Not surprisingly, inadequate social and maternal behaviours are highly correlated with developmental and health deficits in the females and their offspring (Fitzgerald et al. 2020). Additionally, motherhood, particularly for primiparous females, represents a challenging period for the mothers themselves. Despite its importance, many studies have focused on the long-lasting consequences to the offspring while neglecting the neurobehavioral changes in the mothers (Hillerer et al. 2014; Kundakovic and Champagne 2015; Pawluski et al. 2022; Navarro-Moreno et al. 2022; Hoekzema et al. 2022).

The maternal brain goes through hormonal and psychological adaptations during motherhood. The period of ‘matrescence’ will prepare the individual for the stage of motherhood, characterised by a change in behaviour (Orchard et al. 2023). For instance, female rodents start building nests and showing signs of aggression towards conspecifics by the end of their pregnancy (Mann and Svare 1982; Mayer and Rosenblatt 1984; Martín-Sánchez et al. 2015a; Navarro-Moreno et al. 2022). Similarly, the postpartum period is characterised by an increased load of caring-related tasks (e.g., nurturing and grooming the offspring) and shifted interactions with other adult conspecifics, such as the change from attraction towards males to maternal aggression (Martín-Sánchez et al. 2015a; Leuner and Sabihi 2016; Pawluski et al. 2022; Navarro-Moreno et al. 2022). These behaviours are mediated by neuroplastic adaptations in the brain of the females during both pregnancy and postpartum, which are dynamically adjusted to the needs of the offspring (Fleming et al. 1994; Salais-López et al. 2020; Martínez-García et al. 2021). Thus, motherhood represents a stage of long-lasting neuroplastic adaptation in the female brain with potential recurrence during their adult life (Bridges 2016).

An evolutionary conserved architectural framework seems to orchestrate the social brain functioning in all mammals studied, the so called “socio-sexual behaviour network”, including mainly amygdaloid, septal and hypothalamic areas (Newman 1999; Swain and Ho 2019). In the maternal brain, the olfactory bulbs (OB) and hippocampus, as neurogenic areas, have also been shown to play a relevant role (Leuner and Sabihi 2016). Adult neurogenesis, the production of new neurons from adult neural precursors, has emerged as key contributor to parental neuroplasticity in this circuitry. This process occurs mainly in the sub-granular zone (SGZ) of the hippocampal dentate gyrus (DG) and the ventricular-subventricular zone (V-SVZ), which gives rise to the neuroblasts that migrate via the rostral migratory stream (RMS) towards the OB, where they disperse and integrate as inhibitory interneurons (Doetsch and Alvarez-Buylla 1996; Leuner and Sabihi 2016). Adult neurogenesis in these niches is sensitive to hormonal changes occurring during pregnancy and lactation and has been involved in the expression of maternal behaviours (Shingo et al. 2003; Furuta and Bridges 2005; Pawluski et al. 2009; Mak and Weiss 2010; Leuner and Sabihi 2016; Medina and Workman 2020). Interestingly, a recent study showed that pregnancy stimulates adult neurogenesis in the OB, contributing to pup recognition (Chaker et al. 2023).

Mitral cells of the OB project to the piriform cortex (Pir), a large structure of three-layered paleocortex mainly involved in olfactory perception and learning (Stettler and Axel 2009; Martínez-García et al. 2012; Chen et al. 2022). Studies in different species indicate that doublecortin (DCX), a protein expressed by immature neurons, can be observed in layer II of the Pir (Nacher et al. 2002; Rubio et al. 2016; La Rosa et al. 2020), even though this is a non-proliferative area. Indeed, these immature neurons are born during embryonic development, around E14-15 in mice and rats (Gómez-Climent et al. 2008; Rubio et al. 2016), and remain in a posed neuroblast-like state (arrested maturation), slowly maturing with age (Ghibaudi et al. 2023b), and integrating as glutamatergic neurons (Rotheneichner et al. 2018; Benedetti et al. 2020). Some studies have shown that the maturation rate of DCX cells can be affected by olfactory deprivation (Gómez-Climent et al. 2011), stress (Nacher et al. 2004; Abellán-Álvaro et al. 2024) or pharmacological manipulations (Coviello et al. 2020). However, whether motherhood affects this population is unknown.

Thus, here we sought to investigate the potential influence of the first pregnancy on DCX-ir cell density in the Pir. We first assessed the effect of transition to adulthood by comparing prepubescent vs. young adult female mice with full reproductive capacity (Arellano et al. 2024). Secondly, we investigated the effect of pregnancy by comparing young adult virgin vs. pregnant mice, and the effect of lactation by comparing pup-sensitized virgins vs. lactating dams (Martín-Sánchez et al. 2015b). We hypothesised that both transition to adulthood and motherhood would promote maturation, which could be translated as a decrease on the number of immature embryonically-generated DCX-ir neurons at Pir.

## Materials and methods

### Animals

We used 42 female mice (*Mus musculus*) from the CD1 strain. Their age at the time of sacrifice was 4 weeks old for the prepubescent animals, and 12–13 weeks old for the young adult virgin, pregnant, pup-sensitized virgin and dam groups. Subjects were housed in age-matched homologous pairs to avoid isolation stress and kept in polypropylene cages at ~ 24 °C under a 12-h light/dark hour cycle (lights on at 8:00 am) with *ad libitum* access to water and food.

Animal procedures were approved by the Committee of Ethics and Animal Experimentation of the Universitat Jaume I, performed in accordance with the European Union Council Directive of June 3rd, 2010 (6106/1/10 REV1), and under an animal-usage license issued by the Direcció General de Producció Agrària i Ramaderia de la Generalitat Valenciana (code 2015/VSC/PEAI00055 type 2).

### Experimental design

Subjects were randomly assigned to the different experimental groups. For Experiment 1, we used prepubescent (*n* = 7) vs. young adult virgin females (*n* = 7). For Experiment 2, we analysed used virgin (*n* = 8) vs. pregnant (gestational day 17, *n* = 7) females. This samples were parallel series of the samples used elsewhere (Navarro-Moreno et al. 2020). Finally, for Experiment 3 we used pup-sensitised virgins (*n* = 6) vs. lactating dams (postpartum day 4, *n* = 7). For this experiment, females of 10 weeks old were paired with a stud male for three days. When pregnancy was confirmed, each pregnant female was housed with an age-matched virgin female, which remained with the dam since then until the completion of the study at 13 weeks of age. This procedure allows the virgin females to be pup-sensitized, thus controlling that any changes seen in dams were due to motherhood and not to mere contact with pups (Martín-Sánchez et al. 2015b).

In all cases, at corresponding age, females were deeply anaesthetised with an intraperitoneal (i.p.) injection of sodium pentobarbital (Vetoquinol, Madrid, Spain) at 0.02 mg per g of body weight, and transcardially perfused with saline (0.9%) 4% paraformaldehyde (PFA) in 0.1 M phosphate buffer (PB), pH 7.4.

### Tissue processing

After perfusion, brains were carefully extracted from the skull, post-fixed overnight at 4 °C with similar fixative solution and kept in a cryopreserving solution of 30% sucrose in 0.01 M PB at 4 °C until sinking. Five parallel series of 40 μm-thick free-floating coronal sections were obtained from each brain using a freezing microtome (Microm HM-450, Walldorf, Germany). Sections were kept in 30% sucrose in 0.1 M, pH 7.6, at − 20 °C until use.

### Immunofluorescence

We used one out of the five brain series from each subject. Sections corresponding to each Experiment were simultaneously processed using same batches of reagents and antibodies to ensure minimal procedural biases. Immunofluorescence was performed in free-floating conditions. First, endogenous fluorescence was blocked by incubating the sections in 1% NaBH_4_ (sodium borohydride) in 0.05 M tris-buffered saline (TBS) for 30 min at room temperature (RT). Next, to block nonspecific binding, sections were incubated in a 3% mixture of normal donkey serum (NDS) and normal goat serum (NGS) in 0.3% Tx100 in 0.05 M TBS, pH 7.6, for one hour at RT. Then, sections were incubated overnight with the primary antibody against DCX (Guinea pig anti-DCX, 1:4000; EMD Millipore Corp., AB2253), or a mix of DCX and NeuN (Mouse anti-NeuN, 1:2500; EMD Millipore Corp., MAB377), diluted in 0.05 M TBS, pH 7.6, with 2% NDS/NGS in 0.3% Tx100 at 4 °C in agitation. Following day, sections were incubated with the secondary antibodies (1:200 each; Alexa Fluor 488-conjugated goat anti-guinea pig, Molecular Probes, for DCX; Rhodamine Red^TM^-X-conjugated donkey anti-rabbit, Jackson ImmunoResearch, for NeuN) diluted in 0.05 M TBS, pH 7.6, with 2% NDS in 0.3% Tx100 for 90 min at RT. When required, samples were thoroughly rinsed between steps in TBS.

To fluorescently label nuclei, sections were also incubated with 600 nM DAPI (4’,6’-diamino-2-feniindol) in TBS for 45 s at RT. Finally, sections were mounted in glass slides using 0.2% gelatine in TB and cover-slipped with fluorescent mounting medium (FluorSave Tm Reagent; Dako, Glostrup, Denmark).

### Image acquisition and quantification

In all Experiments, we quantified the number of DCX-ir cells in 3–4 representative sections of the Piriform cortex, following Paxinos and Franklin (2012), broadly corresponding to the anterior Pir (Bregma + 1 mm and 0 mm), and middle to posterior Pir (Bregma − 1 and − 2 mm) (Chen et al. 2022; Ghibaudi et al. 2023b). Pictures were obtained by an experimenter blind to the experimental groups from both hemispheres at selected levels centred at layer II. All images were taken using digital DFC495 camera attached to a Leitz DMRB microscope (Leica, Wezlar, Germany). Unless stated otherwise, pictures were taken at 20x magnification. Positive DCX-ir cells at Pir (layer II, occasionally migrating towards layer III) were counted by using the multi-point tool of ImageJ analysis software. The main diameter of these DCX-ir cells in the layer II of the Pir was estimated for a posterior classification between simple, mainly unipolar or bipolar cells (main diameter < 11 μm, more immature) or complex, mainly multipolar cells (main diameter ≥ 11 μm, in a more advanced maturation stage), similarly to what has been done in other studies (Coviello et al. 2021; Ghibaudi et al. 2023b). First, results were averaged and expressed as number of positive cells per mm^2^. In addition, we explored possible differences in the selected Bregma levels, since both the percentage of simple and complex DCX cells (Ghibaudi et al. 2023b) and the connectivity and function of the Pir (Chen et al. 2022) vary according to the antero-posterior axis.

We further analysed DCX-NeuN immunofluorescence in Experiment 3. To study co-localization, representative images of the Pir at the selected levels were taken with a confocal microscope (ZEISS LCS 980) at 20x magnification. Quantification of the percentage of DCX-ir that co-expressed NeuN-ir was conducted manually throughout the whole II layer of the Pir available in each image. Similarly, the main diameter of each DCX-ir cell in layer II, as well as their number of neurites, were determined manually. Figures were processed using Fiji software (NIH) and Adobe Photoshop CS6 (Adobe Systems, MountainView, CA, USA).

### Statistical analysis

Data were analysed using the software IBM SPSS Statistics 26.0 and R. Normality was determined using the Shapiro-Wilk tests. We applied a student’s t-test analysis for independent samples or a repeated measures ANOVA when appropriate, and if data violated normality, we used a Mann-Whitney test. Significance was set al *p* < 0.05. Graphs were created using ggplot2 package (Wickham 2016) in R (version 4.2.1; (R Team 2023).

## Results

**Fig. 1:**
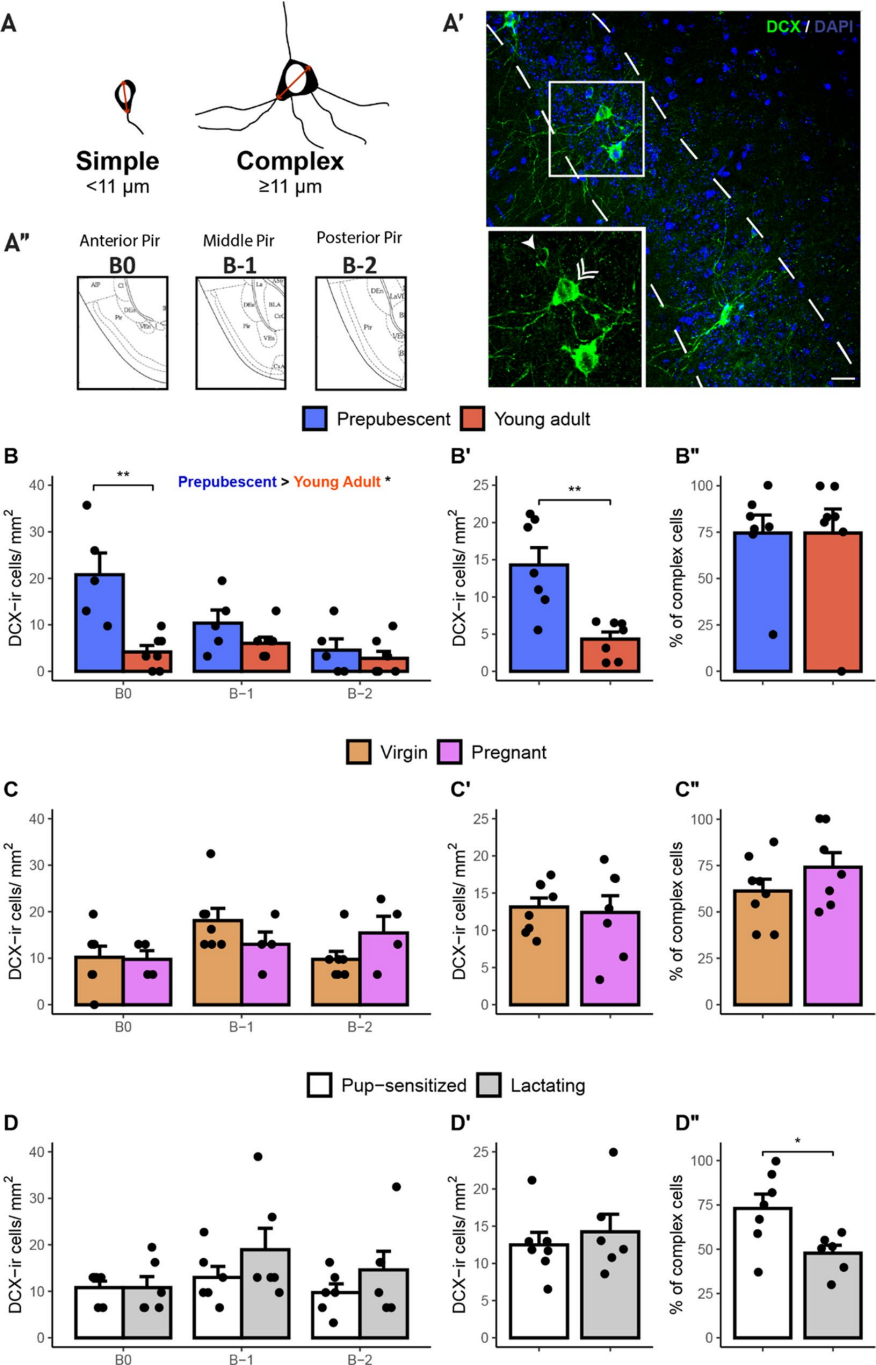
DCX-ir cells at the piriform cortex throughout age and reproductive stages. **A**) Sketch of simple and complex DCX-ir cells depicted in **A’**) representative images, and squared magnification, of DCX immunoreactivity (green) and DAPI staining (blue) in the Pir, with simple (single arrow) and complex (double arrow) DCX-ir cells in Pir’s layer II (dashed lines). Scale bar: 20 μm. **A’’**) Sketch of the Pir at the different Bregma levels analysed. (adapted from Paxinos and Franklin 2012). Transition to adulthood (prepubescent -blue- vs. young adult -red) resulted in a significant decrease of DCX-ir cells in the anterior Pir (**B**), and in the average density of DCX-ir cells in the Pir (**B’**), but it did not affect the percentage of complex cells among total DCX-ir cells (**B’’**). By contrast, pregnancy (virgin -orange- vs. pregnant -pink, **C**), and lactation (pup-sensitized– white- vs. lactating -grey, **D**) did not affect the density of DCX-ir cells through the different regions of its antero-posterior axis (**C**, **D**) or their average density (**C’**, **D’**). The percentage of complex among total DCX-ir cells was decreased in lactating females as compared to pup-sensitized virgins (**D’’**). All graphs represent mean + Standard Error Mean (SEM), and individual values (dots). *: *p* < 0.05; **: *p* < 0.01

As expected, we found DCX-ir labelled cells in layer II of the Pir in all the analysed samples. These cells could be identified as simple (main diameter < 11µm) or complex cells (main diameter ≥ 11µm), being the latter in a more advanced phase of maturation (Fig. 1A, A’). We examined the presence of DCX-ir cells in a priori selected antero-posterior regions of the Pir (Fig. 1A’’), finding that the most anterior part inspected (Bregma + 1 mm) displayed DCX-ir cells in prepubescent females only, and not in any of the analysed samples of older females. Thus, we did not consider this level for further statistical analysis.

In Experiment 1, comparing prepubescent females with young adult females, a repeated measures ANOVA revealed a significant effect of the within subject factor Bregma level (F = 9.7, p = 0.005), the between subject factor group (F = 10.356, p = 0.009) and their interaction (F = 7.8, p = 0.01). Post-hoc pairwise comparisons revealed that differences between groups were strongly significant at the anterior Pir (Bregma 0 mm) (F = 15.7, p = 0.003), and that significance was progressively lost at the middle (Bregma − 1 mm) (F = 3.8, p = 0.074) to posterior regions (Bregma − 2 mm) (F = 0.3, p = 0.6) (Fig. 1B). We then collapsed the data from the three Bregma levels and found a significant decrease in the average density of DCX-ir cells (W = 45.5, p = 0.009; Fig. 1B’). However, the percentage of complex cells DCX-ir cells did not significantly differ with age, suggesting a uniform maturation process (W = 21; *p* = 0.7; Fig. 1A and B’’).

By contrast, in Experiment 2, we did not find any significant difference when comparing virgin and pregnant mice in the density of DCX-ir cells in the layer II of the Pir taking into account the different Bregma levels (Bregma, F = 2.9, p = 0.08; Group, F = 0.0002, p = 0.99; Group x Bregma, F = 2.7, p = 0.09; Fig. 1C). The same was true when analysing the average density of DCX-ir (t = 0.3, p = 0.77; Fig. 1C’) and the percentage of complex DCX-ir cells (t = -1.28, *p* = 0.22; Fig. 1C’’).

Similarly, in Experiment 3 we could not find any significant change in the density in the different Bregma levels (all p > 0.1; Fig. 1D), or the average density of DCX- ir cells (t = 0.62, p = 0.28; Fig. 1D’). This time, however, we found a significant decrease in the average percentage of complex DCX-ir cells (t = -2.63, *p* = 0.012; Fig. 1D) in lactating mice as compared to pup-sensitized virgins.

**Fig. 2:**
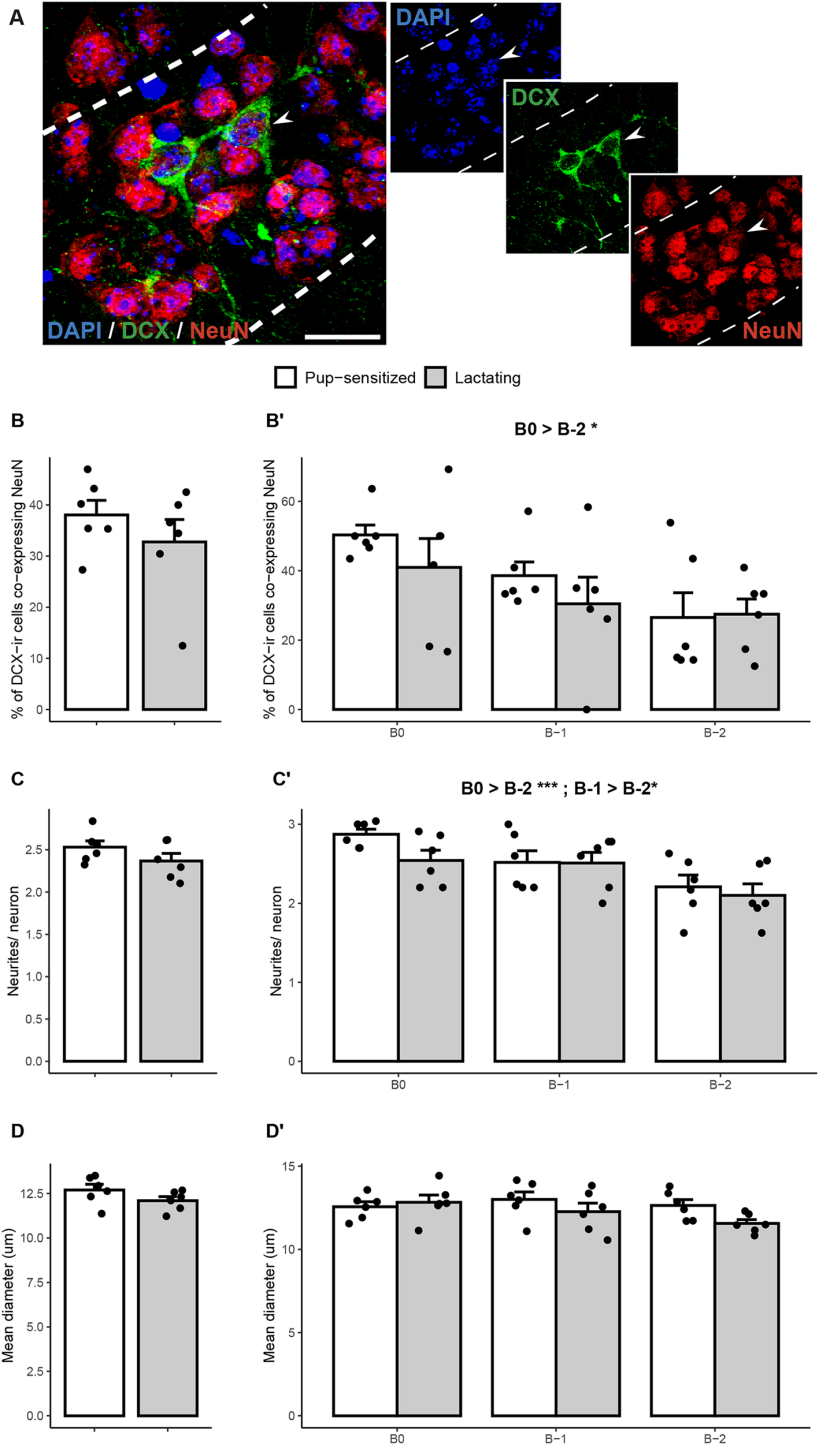
DCX-ir co-expression with NeuN is not affected by lactation in the piriform cortex. **A**) Representative images of DCX (green) and NeuN (red) immunoreactivity and DAPI staining (blue) in the layer II of the Pir, with an example of double-labelled cell (arrow). Scale bar: 10 µm. We did not find significant differences between pup-sensitized (white) vs. lactating in females (grey) on the percentage of **B**) double-stained cells, **C**) number of neurites per DCX-ir cell, and **D**) main diameter of DCX-ir cells. We found a significant effect of Bregma in the percentage of double-stained cells and the number of neurites per neuron (**B’**, **C’**) irrespective of the group. All graphs represent mean + Standard Error Mean (SEM), and individual values (dots)

Since the decrease in the percentage of complex cells could point towards an accelerated disappearance of DCX in complex cells closer to maturation, or a delay in achieving mature features in lactating mice as compared to pup-sensitized virgins, we next explored the co-labelling of DCX with NeuN in these groups. As expected, we found that some of the DCX-ir neurons co-expressed NeuN, supporting their transition to mature neurons (Fig. 2A). However, the average percentage of these double-labelled cells was not significantly different between virgin and lactating mice (t = 1.01, p = 0.34; Fig. 2B). We also assessed other features related to maturation, such as the number of neurites and the size of the soma, but we did not find significant differences between groups on those measures (number of neurites, t = 1.41, p = 0.19; Fig. 2C, main diameter t = 1.52, p = 0.16; Fig. 2D). Interestingly, when we analysed these parameters across the antero-posterior levels, we found a significant effect of Bregma on the percentage of double-labelled cells (F = 4.5, p = 0.024; Fig. 2B’) and the number of neurites (F = 8.1, *p* = 0.003; Fig. 2C’), but not in the diameter (F = 1.8, *p* = 0.2; Fig. 2D). The effect of Group or the interaction between Group and Bregma were not significant in any of the measures (all *p* > 0.1). Accordingly, both the percentage of DCX-NeuN immature neurons and the number of neurites of DCX cells were lower in the posterior Pir, irrespectively of the group.

## Discussion

Our study is in agreement with previous findings showing that age leads to a decrease in the density of DCX-ir cells in the Pir, that lose immaturity markers as they differentiate into glutamatergic neurons. In addition, we found that DCX-ir was first lost within the anterior Pir, and, concomitantly, features in DCX cells associated to maturation (i.e. co-expression with NeuN, higher number of neurites) were increased in this region. Finally, we could not find a strong significant effect of the first pregnancy in the density of these cells.

Motherhood has been previously associated with neuroplastic changes including adult neurogenesis at the V-SVZ which, via the canonical pathway, migrate to the OB and integrate as mature neurons (Shingo et al. 2003; Leuner and Sabihi 2016; Medina and Workman 2020). These plastic changes are adaptive since they are associated with novel odour learning, including pup recognition and maternal care, hence increasing the potential of the offspring to survive (Hillerer et al. 2014; Chaker et al. 2023). Given the olfactory and integrative functions of the Pir, as well as its connections and its reported adaptive capacities (Chen et al. 2022), we expected that a considerably strong adaptive process like ‘matrescence’ would also result in an increased maturation and integration of this population of cells in the Pir. However, in disagreement with our hypothesis, we did not find a significant impact of pregnancy or lactation on the density of these cells, and just a slight change in the percentage of complex DCX-ir cells in lactating females, suggesting a minor loss of complex cells or, alternatively, a slight delay in achieving mature features.

The population of DCX-ir cells at the Pir has been previously described as a niche for protracted neural maturation, where DCX-ir cells decrease with age as they mature and integrate into the neural circuit (Gómez-Climent et al. 2008; Rubio et al. 2016; Rotheneichner et al. 2018; La Rosa et al. 2020; Ghibaudi et al. 2023b). In our study, we found a sharp decline in the density of DCX-ir cells in young adult virgins (roughly 3 months old) as compared to prepubescent females (1 month old), suggesting an important effect of pubertal development in the maturation rate of DCX-ir cells. Albeit following the same trend, a previous study failed to demonstrate a significant decrease in the density of DCX-ir cells until older adulthood, i.e., when comparing 1 month old vs. 12 months old mice (Ghibaudi et al. 2023b). That study was performed in C57/BL6 male mice, so factors such as the sex of the animals, the strain and the method of quantification might account for the observed differences in the time course of maturation.

Interestingly, when considering the different anteroposterior regions, we found that, at the most anterior level inspected (roughly, Bregma + 1 mm), DCX-ir cells were present only in prepubescent animals. In the following level of the anterior Pir (roughly, Bregma 0 mm), the density of DCX-ir cells was significantly different between prepubescent and young adult mice. This difference showed a slight trend in the middle Pir, and was not significantly different in the posterior Pir between the two groups (Fig. 1B). This pattern, together with the data from young adult virgins and lactating females, in which we found a lower co-expression of DCX-NeuN and lower number of neurites in the immature neurons of the posterior Pir, is consistent with a gradient of maturation in which DCX-ir cells mature progressively from anterior to posterior regions. In addition, Ghibaudi et al. (2023b) found a higher density of DCX-ir cells in middle and caudal regions than in anterior ones, a pattern that might also be consistent with an anterior to posterior gradient of maturation. In this context, the anterior Pir receives denser projections from the mitral cells of the OB than the posterior Pir, so anterior Pir might be regarded as primary sensory cortex, whereas the posterior Pir is more linked to associative functions (Chen et al. 2022). Thus, one could hypothesise that this pattern of maturation might be related to a previously described earlier maturation of sensory areas as compared to associative regions (Gogtay et al. 2004), and a later loss of plasticity in associative circuits as compared to sensory ones (Poo and Isaacson 2007). However, future experiments are needed to demonstrate this hypothesis.

More surprising was the absence of statistically significant motherhood-induced differences in DCX expression in the Pir. We hypothesise that these females being primiparous might be an important factor leading to lack of a significant effect. In the DG of the hippocampus, the pattern of cell proliferation depends on both the reproductive state and the reproductive experience and slightly changes depending on whether the mother is primiparous or multiparous (Pawluski and Galea 2007). Therefore, in the Pir, repetition of ‘matrescence’ cycles might result in a stronger, and quantifiable, effect. In addition, the pattern of DCX-ir cell maturation in the Pir is different than that of the V-SVZ or the DG of the hippocampus, with DCX-ir immature neurons of the Pir having an embryonic origin and lacking proliferative capacity (Gómez-Climent et al. 2008; Rubio et al. 2016; Rotheneichner et al. 2018). These different patterns of generation and/or maturation might explain why we have not seen an apparent effect of pregnancy and lactation in the density of DCX-ir cells. Alternatively, it is possible that the time at sacrifice, postpartum day 4, might be too early to see significant differences in the density of a population not affected during pregnancy. In this sense, we detected a slight effect on the composition of DCX-ir population, namely a decrease in the percentage of complex DCX-ir cells, the ones in a more advanced status of maturation. Of note, pregnancy-induced increase in neurogenesis in the OB is transient, with new-born granular cells disappearing by weaning of the litters (Chaker et al. 2023). It is possible then that only a small amount of complex DCX cells might be recruited on the first pregnancy, hence masking any significant effect on the average density of the whole population.

We have identified some potential caveats. Firstly, despite being considered a microtubule-associated protein with functions on differentiation and migration of neurons (Francis et al. 1999; Gleeson et al. 1999; Friocourt 2003), DCX could be involved in other processes which might be related to neuronal development (Yap et al. 2012) or not (Dhaliwal et al. 2016). Thus, interpretations on neuronal maturation based on DCX-ir alone should be done with caution. Secondly, different commercial antibodies against DCX might yield variations on data depending on the species and cerebral area studied (Ghibaudi et al. 2023a), a source of inconsistency that should also be accounted for. In addition, one should acknowledge the possible effects that pair-housing could had in Experiments 2 and 3, were pregnant and lactating females were co-housed with virgin controls. Finally, the findings reported here are just descriptive. Future studies using viral labelling or genetically modified mice which will allow for traceability of DCX neurons, multiple pregnancies and later time points of the lactating process are needed to understand the dynamics of maturation, integration and its functional significance.

Many studies have focused on the effect of motherhood to the offspring while failing to look for the consequences to the mothers themselves. Different imaging techniques have allowed identification of structural and functional neuroplastic adaptations associated with motherhood in humans (Hoekzema et al. 2017; Servin-Barthet et al. 2025). However, these studies can only provide limited data and do not allow for cellular-type identification. Here, we have used a rodent model to study how the population of immature neurons of the Pir is affected by motherhood. Apart from replicating and extending previous findings showing a sharp decrease of DCX-ir neurons in the Pir after puberty, following an anteroposterior gradient, we have found that pregnancy and lactation do not significantly alter the maturation and/or integration of these DCX-ir immature neurons, maybe due to the number of reproductive experiences and/or Pir’s peculiarities in terms of maturation. Therefore, additional maturation inputs, potentially related to olfaction, should be studied to decipher how and why this population, which remains in an immature state, starts to mature.

## Data Availability

The data that support the findings of this study are available from the corresponding author upon request.
